# Identification of Shiga-Toxin-Producing Shigella Infections in Travel and Non-Travel Related Cases in Alberta, Canada

**DOI:** 10.3390/toxins13110755

**Published:** 2021-10-25

**Authors:** Shuai Zhi, Brendon D. Parsons, Jonas Szelewicki, Yue T. K. Yuen, Patrick Fach, Sabine Delannoy, Vincent Li, Christina Ferrato, Stephen B. Freedman, Bonita E. Lee, Xiao-Li Pang, Linda Chui

**Affiliations:** 1 The Affiliated Hospital of Medical School, Ningbo University, Ningbo 315000, China; zhishuai@nbu.edu.cn; 2 School of Medicine, Ningbo University, Ningbo 315000, China; 3 Department of Laboratory Medicine and Pathology, University of Alberta, Edmonton, AB T6G 2B7, Canada; brendonp@dal.ca (B.D.P.); jszelewi@ualberta.ca (J.S.); yyuen@alumni.ubc.ca (Y.T.K.Y.); Xiao-Li.Pang@albertapubliclabs.ca (X.-L.P.); 4 Agency for Food, Environmental and Occupational Health and Safety (ANSES), Food Safety Laboratory, COLiPATH Research Unit & IDPA Genomics Platform, FR-94700 Maisons-Alfort, France; patrick.fach@anses.fr (P.F.); sabine.delannoy@anses.fr (S.D.); 5 Alberta Precision Laboratories-ProvLab, Edmonton, AB T6G 2J2, Canada; vincent.li@albertaprecisionlabs.ca; 6 Alberta Precision Laboratories-ProvLab, Calgary, AB T2N 4W4, Canada; christina.ferrato@albertaprecisionlabs.ca; 7 Alberta Children’s Hospital, Division of Pediatric Emergency Medicine and Gastroenterology, Cumming School of Medicine, University of Calgary, Calgary, AB T2N 1N4, Canada; stephen.freedman@ahs.ca; 8 Alberta Children’s Hospital Research Institute, Department of Emergency Medicine, Cumming School of Medicine, University of Calgary, Calgary, AB T2N 1N4, Canada; 9 Department of Pediatrics, Faculty of Medicine & Dentistry, Women and Children’s Health Research Institute, Stollery Children’s Hospital, University of Alberta, Edmonton, AB T6G 1C9, Canada; bonitlee@ualberta.ca

**Keywords:** *Shigella flexneri*, Shiga toxin, phage, phylogenomic, global transmissions

## Abstract

It has long been accepted that Shiga toxin (Stx) only exists in *Shigella dysenteriae* serotype 1. However, in recent decades, the presence of Shiga toxin genes (*stx*) in other *Shigella* spp. have been reported. We screened 366 *Shigella flexneri* strains from Alberta, Canada (2003 to 2016) for *stx* and 26 positive strains were identified. These isolates are highly related with the majority originating from the Dominican Republic and three isolates with Haiti origin. Both phylogenetic and spanning tree analysis of the 26 Alberta and 29 *stx* positive *S. flexneri* originating from the U.S., France, Canada (Quebec) and Haiti suggests that there are geographic specific distribution patterns (Haiti and Dominican Republic clades). This study provides the first comprehensive whole genome based phylogenetic analysis of *stx* positive *S. flexneri* strains as well as their global transmission, which signify the public health risks of global spreading of these strains.

## 1. Introduction

*Shigella* is a genus of Gram-negative bacterium that can be transmitted to humans through contaminated food, water, or direct/indirect contact with an infected person. The natural reservoirs of *Shigella* are typically human and non-human primates, although *Shigella* infections have been reported in other animals, e.g., chicken and calves [1,2,3,4]. Shigellosis can result from a relatively low infective dose between 10 to 100 organisms and the patient can exhibit symptoms such as diarrhea, fever, stomach pain, nausea, and vomiting [5,6]. In the past century, Shigellosis has decreased greatly through improved sanitation. However, currently *Shigella* is still one of the most important foodborne pathogens both in developing [6] and developed countries [7,8]. It was estimated that *Shigella* causes 188 million cases of disease and 164,300 deaths per year globally [5].

The genus *Shigella* is comprised of four major species including *Shigella dysenteriae*, *Shigella flexneri*, *Shigella sonnei*, and *Shigella boydii*. A large number of virulence factors have been identified in *Shigella* spp. [9], including Shiga toxin (*Stx*) which is commonly found in *S. dysenteriae* serotype 1 and closely resembles Stx in Shiga toxin-producing *Escherichia coli* (STEC). In STEC, Stx can be categorized into two main groups, Stx1 and Stx2. Once it enters the host cells, the A subunit can remove an adenine from 28S rRNA, and therefore, inhibit protein synthesis resulting in cell death [10,11]. Based on DNA sequence and biological activity, *stx*_1_ has three subtypes *stx*_1a_, *stx*_1c_, and *stx*_1d_ while *stx*_2_ has seven subtypes, *stx*_2a_ to *stx*_2g_ [12].

In recent decades, clinical isolates of Stx carrying *Shigella* strains from other *Shigella* spp. were continuously observed. The first published case of stx positive non-*S. dysenteriae* serotype 1 strain was reported in Germany [13], where a *S. sonnei* strain was isolated from a patient with recent travel to Ukraine. To date, stx positive *S. sonnei*, *S. flexneri*, and *S. dysenteriae* serotype 4 had been identified in patients from countries such as the U.S. [14,15,16,17,18], Canada [19], Germany [13], Hungary [20], France [21], Finland [22], and Haiti [23]. At present, only one Canadian province (Quebec) has reported cases of non-*S. dysenteriae* serotype 1 stx positive *Shigella*.

In 2015, an enteric outbreak was declared by Alberta Health Services, AB, Canada and two epidemiologically linked cases were identified to be infected with *Shigella flexneri.* Both cases experienced bloody diarrhea and the molecular testing performed for one of the cases as a result of enrollment to a prospective acute gastroenteritis study [24] (APPETITE) indicated the presence of *stx* genes from the stool sample. Further submission of the stool samples from the two cases by public health resulted in the isolation of *Shigella flexneri* and both strains confirmed to carry the *stx* gene and the expression of the toxin was also confirmed by immunoassay. Consequently, this study was initiated, and the objectives were to screen for Shiga toxin positive isolates from *Shigella flexneri* collected from 2003 to 2016 as related to confirmation and typing of clinical isolates, reference testing service provided by Provincial Laboratory for Public Health (ProvLab) in Alberta, Canada and to further characterize these strains.

## 2. Results

### 2.1. stx_1_-Producing S. flexneri Identified in Clinical Isolates

The *stx*_1_ gene was identified in 26/366 (S1 to S26) *S. flexneri* isolates (Table 1) archived in Alberta from 2003 to 2016. All 26 *S. flexneri* isolates carried the *stx*_1a_ gene subtype and expressed the toxin as detected by the *SHIGA TOXIN QUIK CHEK*™ assay.

### 2.2. Sporadic Cases of stx_1_ Positive S. flexneri in Alberta, Canada

Among the 366 cases infected with *S. flexneri* with isolates archived in Alberta, 76 had travelled to the Caribbean area such as Cuba, Haiti, Dominican Republic, Mexico, El Salvador; 15 of the 26 *stx*_1_ positive *S. flexneri* cases were travel-related and they all had recent travelled to the Dominican Republic except one who had visited the Turks and Caicos Islands. The remaining 11 cases had no recent travel and also had no knowledge of contact with persons who have had recent travel history.

### 2.3. Location of stx_1_ Gene in S. flexneri Strains

In a report of *Shigella* infections related to travel to Hispaniola, it was indicated that the *stx*_1_ gene of these *S. flexneri* was located on a 62 kb φPOC-J13 phage [17]. This phage carries the *stx*_1_ gene and was inserted on the *S. flexneri* chromosome [17]. Whole genome sequence (WGS) analysis showed that the same phage was carried by all of our 26 *stx*_1_ positive *S. flexneri* isolates with a sequence similarity of 99.9% except that strain S1 had a 528 bp deletion and strain S3 had a 180 bp insertion within the phage. In additon, our PCR results of the insertion site showed the φPOC-J13 phage was inserted on the same chromosome site as demonstrated by Gray et al. [17]. All 26 isolates showed 100% sequence identity in the *stx*_1_ gene. The general genome characteristics are shown in Table S3.

### 2.4. Relatedness of Travel and Non-Travel stx_1_ S. flexneri Strains

**Figure 1:**
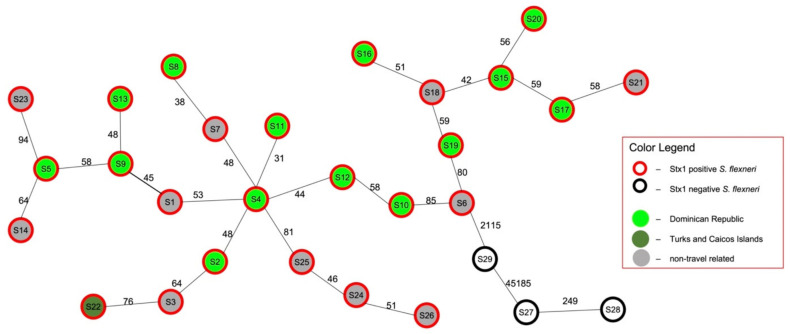
Core genome SNP based minimum spanning tree of 29 *S. flexneri* strains isolated in Alberta, Canada. *S. flexneri* strains were isolated from patients that had recent travel to Dominican Republic (light green in-filled circles), Turks and Caicos Islands (dark green in-filled circles), and non-travel history (grey in-filled circles). The red color of the outer circle represents *stx*_1_ positive *S. flexneri* while the ones with black outer circle are *stx*_1_ negative *S. flexneri*. Numbers on lines indicate core genome SNP differences between adjacent strains.

Core genome-based SNP analysis was performed to evaluate the extent of relatedness between the 26 *stx*_1_ positive *S. flexneri* strains in Alberta. These isolates differed from each other from 31 (S4 and S11) to 170 (S20 and S26) SNPs at the core genome level (3.6 MB) (Table S4) with a median SNP difference of 104. Data from a minimum spanning tree further illustrated that closely related strains with less SNP differences clustered together (Figure 1) regardless of the patient travel history. The three *stx*_1_ negative *S. flexneri* (S27, S28 and S29) clustered separately from the 26 *stx*_1_ positive *S. flexneri* group, and they differ by a distance of >2115 SNPs. A pairwise whole genome analysis was also performed on these 26 strains and genome similarities ranged from 99.3% to 99.7% (Table S5).

### 2.5. Relatedness of stx_1_ Positive S. flexneri Strains from Different Countries

**Figure 2:**
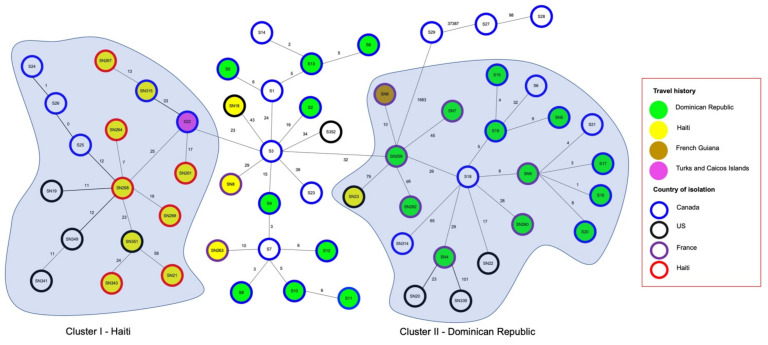
Core genome SNP based minimum spanning tree of *stx*_1_ positive *S. flexneri* strains isolated from four countries and their related travel history. These strains were isolated from patients in Canada (blue outer circles), U.S. (black outer circles), France (purple outer circles), and Haiti (red outer circles). The travel history is depicted by the filled colors of the circles with green (Dominican Republic), yellow (Haiti), light brown (French Guiana), and purple (Turks and Caicos Islands). Country specific Clusters I and II are shaded in light blue. Numbers on lines indicate core genome SNP differences between adjacent strains.

The core genome SNPs analysis on sequences of 55 *stx*_1_ positive (26 from Alberta and 29 from the NCBI genome database) and 3 *stx*_1_ negative *S. flexneri* strains (S27, S28, and S29) were performed to examine the relatedness of Alberta isolates with global isolates from NCBI (Figure 2). The 29 NCBI sequences were from cases diagnosed in the US, France, Canada (Quebec), and Haiti along with their travel histories (Table 1). Interestingly, cases with travel history all indicated to have travelled to or reside in the Caribbean countries, including the Dominican Republic, Haiti, French Guiana, and Turks and Caicos Islands. The pairwise SNP difference analysis (Table S6) demonstrated that the 55 *stx*_1_ positive *S. flexneri* strains were closely related and the SNPs differences varied from 0 (S25 and S26) to 189 (SN21 and SN339) SNPs with a core genome size of 3.6 MB.

In the minimum spanning tree (Figure 2), two country specific clusters (I and II) of *stx*_1_ positive *S. flexneri* were identified based on the case’s country of residency or recent travel destinations. In Cluster I, all strains with known travel history were isolated from cases in or who had visited Haiti except S22, which was isolated from a case who travelled to Turks and Caicos Islands. In comparison, all strains within Cluster II were isolated from cases who travelled to the Dominican Republic, except one that visited Haiti (SN23) and one that visited French Guiana (SN6).

### 2.6. Bayesian Phylogenomic Analysis of stx_1_ Positive S. flexneri from Different Countries

**Figure 3:**
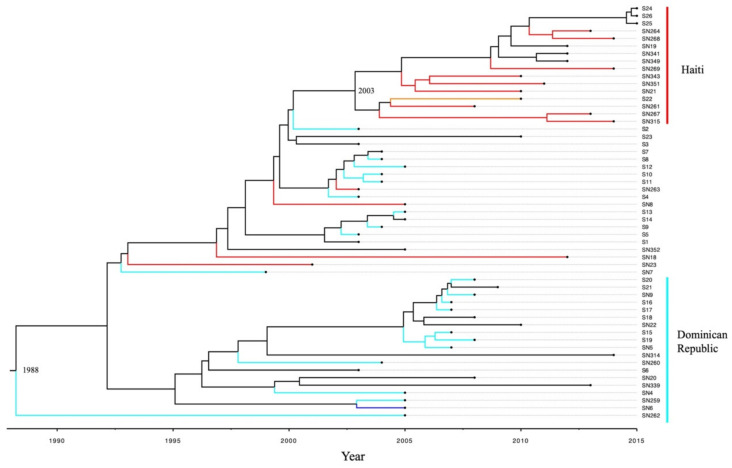
Bayesian phylogenetic tree based on 55 *stx*_1_ positive *S. flexneri* strains. Two country specific Haiti and Dominican Republic were formed. The branches were colored according to the strain’s country of isolation or the respective case’s recent travel country. Branches colored with red represents Haiti strains while cyan represents Dominican Republic strains. The branches colored with orange and blue represent Turks Caicos Island and French Guiana strains, respectively. The most common ancestor of *stx*_1_ positive *S. flexneri* strains emerged in 1988. The Haiti Clade arose in 2003.

In order to investigate the country related evolution of all 55 *stx*_1_ positive *S. flexneri* strains as revealed in the spanning tree analysis, a Bayesian phylogenomic analysis was performed. As illustrated in Figure 3, *stx*_1_ positive *S. flexneri* isolates formed two clusters based on their source countries including Cluster I—Haiti and Cluster II—Dominican Republic. Within Cluster II—Dominican Republic, 16 strains had source country data, among which only two strains [one Haiti (SN23) and one French Guiana (SN6)] were not from the Dominican Republic. In Cluster—Haiti, only one Turks and Caicos Islands strain (SN22) was observed while the other nine strains were all from Haiti. Root reference (Figure 3) suggested that the most common ancestor of the *stx*_1_ positive *S. flexneri* most likely first emerged in 1988 [95% credible interval (CI): 1984–1992] in the Dominican Republic. A Haiti Cluster arose in 2003 with 95% CI of (2001, 2004). Strains S24, S25, and S26 were from Canadian cases with no travel or contact with people with recent travel. However, their isolates grouped within the Haiti Cluster (Figure 2 and Figure 3) suggesting the origin might be from Haiti.

### 2.7. Genome Evolution of stx_1_ Positive S. flexneri Strains

**Figure 4:**
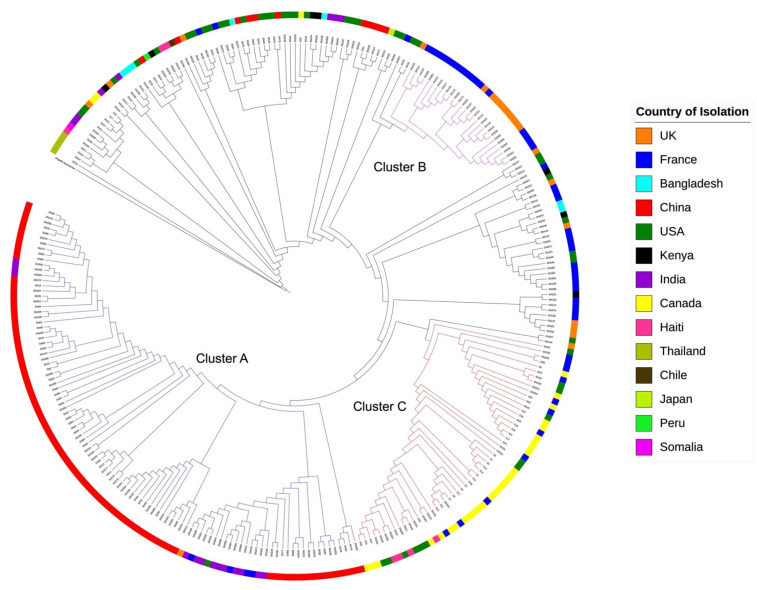
Phylogenetic tree based on 297 *S. flexneri* genomes. *S. dysenteriae* was used as the outgroup and branch length was not represented in this tree. The colors of circle represent 14 different countries where the *S. flexneri* were isolated. Three geographic specific clusters were identified, and their branches were colored in blue, red, and purple respectively. Strains in Cluster A were mostly isolated in China and India. Most of strains in Cluster B were from UK and France. In Cluster C consisted of all *stx*_1_ positive *S. flexneri* strains.

A maximum likelihood phylogenetic tree was constructed (Figure 4) to understand the evolutionary relationship of *S. flexneri*, including *stx* negative and *stx* positive strains. This tree was based on 57,889 SNPs (core genome size of 1.7 MB) identified among the 296 *S. flexneri* genomes originated from 14 different countries. Overall, strains from the same or geographically close countries clustered closely together (Figure 4). For example, in Cluster A, 94.3% of strains came from two Asian countries, China (87.0%, *n* = 86) and India (13%, *n* = 13). It was also observed that Cluster B strains (*n* = 27) were all from the UK and France. All 55 *stx*_1_ positive *S. flexneri* strains clustered in a single clade (Cluster C) in the phylogenetic tree suggesting that the *stx*_1_ positive *S. flexneri* strains are highly related and share a common ancestor.

## 3. Discussion

Evolution of pathogens can give rise to highly virulent strains as illustrated by the 2011 STEC O104:H4 outbreak in Germany. That outbreak strain originated from an enteroaggregative *E. coli* (EAEC) but later acquiring a Shiga-toxin-encoding phage and antibiotic resistance genes making it highly pathogenic [25]. Although uncommon, identification of *stx* positive *S. flexneri* strains have been reported by several studies [17,19]. Therefore, understanding their global circulation and evolution is of great public health importance. This study provides the first comprehensive whole genome-based analysis of *stx* positive *S. flexneri* strains obtained globally.

In this investigation, 26/366 *S. flexneri* strains isolated between 2003 and 2016 in Alberta were found carrying Shiga toxins. Among the 366 *S. flexneri* cases, 23 cases (data not shown) had recent travel to the Dominican Republic and 60.9% (14/23) of the strains isolated from these cases carried the *stx*_1_ gene. A similar study by Gray et al. [23] also found 57.1% (4/7) of *S. flexneri* isolates cultured from cases in Haiti tested positive for *stx*_1_. No severe clinical symptoms such as hemolytic urrmic syndrome were observed for *stx*_1_ positive *S. flexneri* infection as reported [23]. The *stx*_1_ carried by the clinical isolates in this study belong to *stx*_1a_ which is not as virulent as other subtypes, such as *stx*_2a_ [26]. Currently, it is still unknown whether acquisition of this *stx*_1_ phage has increased the pathogenicity of these *Shigella* strains; however, it is evident that *stx*_1_ positive *S. flexneri* has exceptionally high prevalence rates (60.9% observed in Alberta cases returning from Dominican Republic and 57.1% observed in residents in Haiti as shown above) suggesting the *stx*_1_ positive *S. flexneri* strains have established in Alberta and spread locally.

When compared to sequence of *stx*_1_ positive *S. flexneri* strains reported in other countries (Figure 2), we have observed no apparent clustering of strains from the same country of diagnoses. Instead, strains isolated from cases residing in or had recent travel to the same country (i.e., Dominican Republic) clustered closely to each other. For example, two strains (S16 and SN9) were almost identical (1 SNP difference) across their core genome, albeit the cases were diagnosed in Canada and France (Figure 2), and they all have travelled to the Dominican Republic. Results for pairwise whole genome similarity analysis (Table S5) further support that all *stx*_1_ positive *S. flexneri* strains are extremely similar, with genome similarities ranging from 99.3% to 99.7%. This provides strong evidence that most of the Alberta clinical cases caused by *stx*_1_ positive *S. flexneri* were related to international travel to the Caribbean countries highlighting the public health risk of global transmission.

Based on the travel information collected, 12 of the 26 infections in the Canadian cases were not travel-related and had no acknowledgement of personal contact to people returning from the Caribbean countries, suggesting the possibility of secondary transmission in Canada. This has substantial public health implications because of the potential of having carriers of these strains or unidentified source, e.g., imported food, in Alberta. In a similar U.S. study, some Stx-producing *S. sonnei* strains circulating in south California may have been introduced from Mexico but then established themselves locally [10]. Therefore, under appropriate conditions, this endemic Caribbean *S. flexneri* may become endemic in a new geographic region, causing sporadic infections or even outbreaks. Even if the *stx*_1_ positive *S. flexneri* were not establishing endemicity in an area outside of the Caribbean, its phage φPOC-J13 carrying the *stx*_1_ gene can be passed on to the other bacteria thorugh horizonal gene transfer [26] and cause public health problems. The *stx*_1_ carrying φPOC-J13 phages can infect and lysogenize *E. coli* and other *Shigella* strains, such as *S. dysentariae*, *S. boydii*, and *S. sonnei* [17,23,27]. Once brought into a new geographic area by international travelers, this phage may find its way to establish themselves in local pathogenic strains. Under this circumstance, a more pathogenic strain may evolve and cause severe outbreaks similar to the *E. coli* O104 outbreak started in Germany.

Similar to the findings of Fogolari et al. [27], our spanning tree and Bayesian phylogenetic analyses demonstrated that *stx*_1_ positive *S. flexneri* have developed country specific subclades (the Haiti Clade and the Dominican Republic Clade). Haiti and Dominican Republic are located on the same island of Hispaniola whose size is 76,192 km^2^, therefore, the existence of two country-specific clades is unexpected. Although sharing the same island, the two countries have considerable differences with regard to economy, geography, and demography, etc. [28,29]. It has been found that people of different ethnic groups have unique gut microbial profiles which might be caused by their differences in diet, genetics, cultural habits, and socioeconomic status [30]. There is a great difference in the ethnicity of the population between Haiti and Dominican Republic [28,29], and therefore the *S. flexneri* strains from the two country specific clades may have adapted themselves to survive in these two different populations.

Although it has been demonstrated that the φPOC-J13 can integrate in *E. coli* or other *S. flexneri* strain in laboratory conditions [17], all 55 φPOC-J13 carrying *S. flexneri* isolated from cases fall into the same clade on the phylogenetic tree (296 *S. flexneri* strains) but was not observed in other clades of the tree. This result indicates that the φPOC-J13 can only be steadily transferred to certain types of *S. flexneri* populations as bacteria have developed several phage resistance mechanisms to prevent phage transfection [31]. This unique clustering allows us to suspect that the common ancestor of the φPOC-J13 carrying *S. flexneri* strains might have lost their ability to resist φPOC-J13 transfection, which leads to stable integration of φPOC-J13. In the phylogenetic tree (Figure 4), we have also observed clades with abundant Asian *S. flexneri* strains or UK-France strains. This result implicates that the transmission of some *S. flexneri* strains are limited to geographically close regions, which is also the case for various pathogens [32,33,34]. However, this geographical boundary will be diminishing with the global spread of pathogens facilitated by increasing global travel and trades.

## 4. Conclusions

In summary, we have identified 26 *stx*_1_ positive *S. flexneri* strains among 366 clinical *Shigella* isolates from 2003 to 2016 in Alberta, Canada. The limitation of this study is the failure to obtain clinical information of all the *S. flexneri* cases and the outcome of the disease. The majority of the 26 *stx*_1_ positive *S. flexneri* strains originated from Dominican Republic while three of them may have Haiti origin based on genomic anaylsis. These strains were observed to have geographic specific distribution patterns as Haiti and Dominican Republic specific clades. However, with the capability of the transferring of the *stx* gene on the phage to other strains, and with increase in international travel, it can facilitate the global spread and cause an alarm in public health, therefore systematic surveillance of *stx*_1_ positive *S. flexneri* strains is of high importance.

## 5. Materials and Methods

### 5.1. Bacterial Strains

This study included all archived *S. flexneri* (*n* = 366) clinical strains referred to ProvLab for confirmation and typing from 2003 to 2016 in Alberta, Canada. These isolates were retrieved from 10% skim milk stored at −80 °C, cultured on sheep blood agar plates [BAP] (Dalynn Biologicals, Calgary, AB, Canada) at 37 °C for 16 h.

### 5.2. Identification of Shiga Toxin-Producing S. flexneri

Real time PCR amplification of *stx*_1_ and *stx_2_* genes as a multiplex assay was used for *stx* screening as previously described [35]. Sequences of primers and probes for PCR are shown in Table S1. Subtyping of *stx*_1_ positive isolates was performed by conventional PCR as described by Scheutz et al. [12]. The presence of the toxin was confirmed by *SHIGA TOXIN QUIK CHEK*™ (TechLab, Blacksburg, VA, USA), a commercial enzyme immunoassay. The procedure was carried out as per manufacturer’s instruction.

To verify the insertion site of the phage φ POC-J13 [14] into the genome of *stx* positive *S. flexneri*, two sets of PCRs were designed using Primer 3 [36]. Each set has a primer targeting the phage φPOC-J13 region and another primer targeting upstream or downstream of insertion sites based on the *S. flexneri* genome sequence (Figure S1 and Table S1). The final volume of each PCR reaction was 20 μL, containing 10 μL of 2X SYBR^®^ Green PCR Master Mix (Life Technologies, Carlsbad, CA, USA), and 900 nM of each primer. Real-time PCR was performed in an ABI 7500 Fast system (Applied Biosystems, Foster City, CA, USA) with the following conditions: 95 °C for 10 min, 40 cycles of 95 °C for 15 s, 60 °C for 1 min.

### 5.3. Whole Genome Sequencing and Pairwise Whole Genome Similarity Analysis

WGS was performed on all *stx* positive *S. flexneri* strains identified in this study along with three *stx* negative *S. flexneri* strains. Genomic DNA was extracted from overnight cultures using the DNeasy Blood and Tissue Kit (Qiagen, Valencia, CA, USA). Sequencing libraries were prepared using the Nextera XT kit (Illumina, San Diego, CA, USA), and WGS was carried out on the Illumina MiSeq platform. Trimmomatic Version 0.38 [37] was used to trim the low-quality reads of each genome. De novo assembly was performed using SPAdes Version 3.9.1 [38] and contigs smaller than 500 bp were removed. Pairwise whole genome similarity analysis was performed using REALPHY 1.12 [39] where strain S1 was randomly selected as reference.

### 5.4. Core Genome SNP Analysis and Minimum Spanning Tree

The core genome of all *stx* positive *S. flexneri* and three *stx* negative strains were analysed using REALPHY 1.12 [39]. One of the *stx* positive *S. flexneri* strains was randomly selected as reference, and genome sequences of all other strains were then mapped to the reference genome to identify their core genome. The core genome was analyzed by MEGA X Version 10.1.0 [40] to calculate their pairwise SNP differences. In addition, a minimum spanning tree was generated using Phyloviz [41] based on core genome SNPs differences.

Another core genome SNP analysis was performed with an extended number of *S. flexneri* strains by inclusion of sequence data of *stx* positive *S. flexneri* strains (*n* = 29) whose genome sequences were obtained from the NCBI genome database. Their epidemiological data were also collected through NCBI or their corresponding publications. The same analysis settings were used as described in the previous paragraph.

### 5.5. Bayesian Phylogenetic Analysis of stx Positive S. flexneri Strains from Different Countries

Core genomes of all *stx* positive *S. flexneri* strains identified in this study and 29 strains from NCBI database were analyzed using REALPHY 1.12 [39]. Recombination sequences in the core genome were predicted and removed using Gubbins [42]. The core genome SNP alignment was subjected to Bayesian evolutionary analysis using BEAST Version 2.6 [43]. For the BEAST analysis, HKY substitution model, strict molecular clock, constant population size model was selected. The chain length was set to 100 million and sampling was set to every 10,000 iterations. The tip dates were defined as the year of isolation. The BEAST tree was annotated using TreeAnnotater from the BEAST package, visualized using FigTree Version 1.4.4 (http://tree.bio.ed.ac.uk/software/figtree/), and annotated using iTOL [44,45].

### 5.6. Phylogenomic Analysis of S. flexneri

To understand the evolutionary trajectory of these *stx* positive *S. flexneri* isolates, a maximum likelihood (ML) phylogenetic tree was constructed. This analysis included 267 *S. flexneri* genomes downloaded from the NCBI database (Table S2) and sequence of 29 *S. flexneri* strains identified in this study. One *S. dysenteriae* genome (Accession No.: NC_007606.1) from NCBI was used as an outgroup. The core genome was called, and recombination sequences were removed as described in the previous paragraph. The phylogenetic tree was constructed using RAxML Version 8.2.4 with GTRGAMMA option and visualized using the Interactive Tree of Life (iTOL) [44,45].

## Figures and Tables

**Table 1:** *stx*_1_ positive *S. flexneri* strains and associated demographic data.

Strain Name in This Study	Strains Names in NCBI Database	Country Where Shigellosis Was Diagnosed	Travel History	Year of Diagnosis	Strain Name in This Study	Strains Names in NCBI Database	Country Where Shigellosis Was Diagnosed	Travel History	Year of Diagnosis
S1	S1	Canada	NO ^a^	2003	SN20	BS982	USA	NA ^b^	2008
S2	S2	Canada	Dominican Republic	2003	SN21	BS937	USA	Haiti	2010
S3	S3	Canada	NO ^a^	2003	SN22	BS942	USA	NA ^b^	2010
S4	S4	Canada	Dominican Republic	2003	SN23	BS974	USA	Haiti	2001
S5	S5	Canada	Dominican Republic	2003	SN259	BS1023	France	Dominican Republic	2005
S6	S6	Canada	NO ^a^	2003	SN260	BS1022	France	Dominican Republic	2004
S7	S7	Canada	NO ^a^	2004	SN261	BS1025	France	Haiti	2008
S8	S8	Canada	Dominican Republic	2004	SN262	BS1044	France	Dominican Republic	2005
S9	S9	Canada	Dominican Republic	2004	SN263	BS1021	France	Haiti	2003
S10	S10	Canada	Dominican Republic	2004	SN264	BS1057	Haiti	Haiti	2013
S11	S11	Canada	Dominican Republic	2004	SN267	BS1039	Haiti	Haiti	2013
S12	S12	Canada	Dominican Republic	2005	SN268	BS1059	Haiti	Haiti	2014
S13	S13	Canada	Dominican Republic	2005	SN269	BS1060	Haiti	Haiti	2014
S14	S14	Canada	NO ^a^	2005	SN314	SH200	Canada	NA ^b^	2014
S15	S15	Canada	Dominican Republic	2007	SN315	SH199	Canada	Haiti	2014
S16	S16	Canada	Dominican Republic	2007	SN339	BS989	USA	NA ^b^	2013
S17	S17	Canada	Dominican Republic	2007	SN341	BS972	USA	NA ^b^	2012
S18	S18	Canada	NO ^a^	2008	SN343	BS968	USA	Haiti	2010
S19	S19	Canada	Dominican Republic	2008	SN349	BS973	USA	NA ^b^	2012
S20	S20	Canada	Dominican Republic	2008	SN351	BS971	USA	Haiti	2011
S21	S21	Canada	NO ^a^	2009	SN352	BS951	USA	NA ^b^	2005
S22	S22	Canada	Turks and Caicos Islands	2010	SN4	BS1042	France	Dominican Republic	2005
S23	S23	Canada	NO ^a^	2010	SN5	BS1045	France	Dominican Republic	2007
S24	S24	Canada	NO ^a^	2015	SN6	BS1024	France	French Guiana	2005
S25	S25	Canada	NO ^a^	2015	SN7	BS1041	France	Dominican Republic	1999
S26	S26	Canada	NO ^a^	2015	SN8	BS1043	France	Haiti	2005
					SN9	BS1046	France	Dominican Republic	2008
					SN18	BS988	USA	Haiti	2012
					SN19	BS938	USA	NA ^b^	2012

^a^—NO means the patient had no recent travel history; ^b^—NA means the patient’s travel history is not available.

## Supplementary Materials

The following are available online at https://www.mdpi.com/article/10.3390/toxins13110755/s1, Table S1: PCR primers used in this study. Table S2: NCBI accession numbers of *S. flexneri* strains used in this study. Table S3: general genomic characteristics of *S. flexneri* strains. Table S4: SNP differences based on the core genome of 26 *stx* positive *S. flexneri* strains from Canadian cases. Table S5: pairwise genome similarity *stx*_1_ positive *S. flexneri* strains. Table S6: SNP differences based on the core genome of 55 *stx* positive *S. flexneri* strains. Figure S1: location of primers for confirming the specific insertion of phage φPOC-J13 in the chromosome of *S. flexneri*.
