# Supplementary material for: Identification of Shiga-Toxin-Producing Shigella Infections in Travel and Non-Travel Related Cases in Alberta, Canada
Source: Toxins (Basel). 2021 Oct 25;13(11):755. doi: 10.3390/toxins13110755 (PMC8618429; doi:10.3390/toxins13110755)
Supplement: Supplementary file 1 [file toxins-13-00755-s001.zip › toxins-1413879.pdf]

Supplementary Materials: Identification of Shiga-Toxin-Producing Shigella Infections in Travel and Non-Travel Related Cases in Alberta, Canada

Shuai Zhi, Brendon D. Parsons, Jonas Szelewicki, Yue T. K. Yuen, Patrick Fach, Sabine Delannoy, Vincent Li, Christina Ferrato, Stephen B. Freedman, Bonita E. Lee, Xiao-Li Pang and Linda Chui

Table S1. PCR primers used in this study.

Assay	Oligo	Sequence (5'-3')	Amplicon size	Reference:
stx$_{1/2}$ detection	*stx*$_{1/2}$-F	TTTGTYACTGTSACAGCWGAAGCYTTACG	131	[1]
	stx$_{1/2}$-R	CCCCAGTTCARWGTRAGRTCMACRTC		
	stx$_{1}$ probe[a]	6FAM-CTGGATGAT/Z/CTCAGTGGGCGTTCTTATGTAA-NFQ		
	stx$_{2}$ probe[a]	6FAM-TCGTCAGGC/Z/ACTGTCTGAAACTGCTCC-NFQ	128	
φPOC-J13 insertion site validation	PS1-F[b]	TCATCGCAATGGTTACAGGA	642	This study
	PS1-R[b]	CACCCTGAGGTTTTTGAGGA		
	PS2-F[b]	GATGACAAAGGGTGGATTCG	916	
	PS2-R[b]	GCCCATGACCACACAATATG		
stx$_{1}$ subtyping	*stx*$_{1a}$-F	CCTTTCCAGGTACAACAGCGGTT	478	[2]
	stx$_{1a}$-R	GGAAACTCATCAGATGCCATTCTGG		
	stx$_{1c}$-F	CCTTTCCTGGTACAACTGCGGTT	252	
	stx$_{1c}$-R	CAAGTGTTGTACGAAATCCCCTCTGA		
	stx$_{1d}$-F	CAGTTAATGCGATTGCTAAGGAGTTTACC	203	
	stx$_{1d}$-R	CTCTTCCTCTGGTTCTAACCCCATGATA		

a – The ZEN™ internal quencher is denoted in probe sequences by /Z/ between 9th and 10th nucleotides.
b – The PS1 and PS2 refer to the upstream and downstream phage insertion sites.

Table S2. NCBI accession numbers of *S. flexneri* strains included in this study.

Strain Name	NCBI Accession Number	Strain Name	NCBI Accession Number	Strain Name	NCBI Accession Number	Strain Name	NCBI Accession Number
SN1	NC_004337.2	SN87	AZQF00000000.1	SN234	AFHA00000000.1	SN329	AZPS00000000.1
	NC_004851.1	SN88	AZQG00000000.1	SN235	AFHB00000000.1	SN330	AZPU00000000.1
SN2	JMRK00000000.1	SN89	AZQH00000000.1	SN236	AFHC00000000.1	SN331	AZPV00000000.1
SN4	LAIB00000000.1	SN90	AZQI00000000.1	SN237	AFHD00000000.1	SN332	AZPW00000000.1
SN5	LAIE00000000.1	SN91	AZQJ00000000.1	SN238	AFGV00000000.1	SN333	AZPY00000000.1
SN6	LAHY00000000.1	SN92	AZQK00000000.1	SN239	AFGW00000000.1	SN334	AZQA00000000.1
SN7	LAIA00000000.1	SN93	AZQL00000000.1	SN240	AFGX00000000.1	SN335	AZQC00000000.1
SN8	LAIC00000000.1	SN94	AZQM00000000.1	SN241	AFGY00000000.1	SN336	AZQD00000000.1
SN9	LAIF00000000.1	SN104	MTPK00000000.1	SN242	AFGZ00000000.1	SN338	NEDR00000000.1
SN10	LVIJ00000000.1	SN105	UDOV00000000.1	SN251	AMJP00000000.1	SN339	PUGK00000000.1
SN11	LVIX00000000.1	SN106	UINU00000000.1	SN252	AMJT00000000.1	SN340	PUGR00000000.1
SN12	LVIL00000000.1	SN107	UINZ00000000.1	SN253	AMJV00000000.1	SN341	PUGW00000000.1
SN13	LVJA00000000.1	SN108	UDPM00000000.1	SN254	AMJW00000000.1	SN342	PUGY00000000.1
SN14	NIYQ00000000.1	SN109	UDOU00000000.1	SN255	AMJY00000000.1	SN343	PUGZ00000000.1
SN15	NIYR00000000.1	SN110	UIOD00000000.1	SN256	AMKF00000000.1	SN344	PUHA00000000.1
SN16	NIYV00000000.1	SN111	UIOL00000000.1	SN257	ANAN00000000.1	SN345	PUHB00000000.1
SN17	NIYU00000000.1	SN112	UDPP00000000.1	SN258	AMWM00000000.1	SN346	PUHC00000000.1
SN18	PUGL00000000.1	SN113	UDPR00000000.1	SN259	LAHX00000000.1	SN347	PUHD00000000.1
SN19	PUHH00000000.1	SN114	UINW00000000.1	SN260	LAHW00000000.1	SN348	PUHE00000000.1
SN20	PUGQ00000000.2	SN115	UIOB00000000.1	SN261	LAHZ00000000.1	SN349	PUGJ00000000.1
SN21	PUHI00000000.1	SN116	UDPX00000000.1	SN262	LAID00000000.1	SN350	PUGS00000000.1
SN22	PUHG00000000.1	SN117	UDQF00000000.1	SN263	LAHV00000000.1	SN351	PUGX00000000.1
SN23	QDEA00000000.1	SN118	UDPS00000000.1	SN264	LAJS00000000.1	SN352	PUHF00000000.1
SN24	QXIA00000000.1	SN119	UDQE00000000.1	SN265	LAJP00000000.1	SN353	UDOI00000000.1
SN25	QXHZ00000000.1	SN120	UIOC00000000.1	SN266	LAJR00000000.1	SN354	UDOQ00000000.1
SN26	QXIB00000000.1	SN121	UDQD00000000.1	SN267	LAJQ00000000.1	SN355	UEKU00000000.1
SN27	QXHY00000000.1	SN122	UDQK00000000.1	SN268	LAJU00000000.1	SN356	UDOX00000000.1
SN28	QWSQ00000000.1	SN124	UIOZ00000000.1	SN269	LAJV00000000.1	SN357	UDPB00000000.1
SN29	QWSP00000000.1	SN130	UDST00000000.1	SN270	LAJW00000000.1	SN358	UEKY00000000.1
SN30	QWSM00000000.1	SN134	UDTE00000000.1	SN272	LVIO00000000.1	SN359	UDPA00000000.1
SN31	QWRR00000000.1	SN137	UIPA00000000.1	SN273	LVIF00000000.1	SN360	UDPC00000000.1
SN32	QWSA00000000.1	SN145	UDVH00000000.1	SN274	LVIH00000000.1	SN361	UDPF00000000.1
SN33	QWRZ00000000.1	SN147	UDUR00000000.1	SN275	LVIP00000000.1	SN362	UDPI00000000.1
SN34	QWRS00000000.1	SN158	UIPL00000000.1	SN276	LVIQ00000000.1	SN363	UDPJ00000000.1
SN35	QWTR00000000.1	SN189	UIPS00000000.1	SN277	LVIR00000000.1	SN364	UDPN00000000.1
SN36	QWTD00000000.1	SN197	UELP00000000.1	SN278	LVIS00000000.1	SN365	UDPU00000000.1
SN37	QWTB00000000.1	SN199	CP000266.1	SN279	LVIT00000000.1	SN366	UDPQ00000000.1
SN38	QWSN00000000.1	SN200	CP001383.1	SN280	LVIU00000000.1	SN367	UDPO00000000.1
SN39	QWTF00000000.1		CP001384.1	SN281	LVIV00000000.1	SN368	UDPT00000000.1
SN40	QWTE00000000.1		CP001385.1	SN282	LVIW00000000.1	SN369	UDPV00000000.1
SN41	QWST00000000.1		CP001386.1	SN283	LVIY00000000.1	SN370	UDQA00000000.1
SN42	QWSL00000000.1		CP001387.1	SN284	LVIZ00000000.1	SN371	UDPZ00000000.1
SN43	QWSR00000000.1		CP001388.1	SN285	LVIG00000000.1	SN372	UDQS00000000.1
SN44	QWTP00000000.1	SN203	CP026098.1	SN286	LVII00000000.1	SN373	UDQH00000000.1
SN45	QWTC00000000.1		CP026099.1	SN287	LVIK00000000.1	SN374	UDQM00000000.1
SN50	AZOG00000000.1	SN206	CP012735.1	SN288	LVIM00000000.1	SN382	UDSU00000000.1

SN51	AZOH00000000.1		CP012732.1	SN289	LVIN00000000.1	SN388	UDTA00000000.1
SN52	AZOI00000000.1		CP012733.1	SN290	LVJB00000000.1	SN396	UDTK00000000.1
SN53	AZOJ00000000.1		CP012734.1	SN291	LVJC00000000.1	SN405	UDUK00000000.1
SN54	AZOK00000000.1		CP012736.1	SN292	NGVX00000000.1	SN416	UDVE00000000.1
SN55	AZOL00000000.1		CP012140.1	SN293	NGVW00000000.1	SN430	UDVG00000000.1
SN56	AZOM00000000.1		CP012141.1	SN294	NGVZ00000000.1	SN433	UDVO00000000.1
SN57	AZON00000000.1	SN207	CP012142.1	SN295	NGWG00000000.1	SN457	UDWW00000000.1
SN58	AZOO00000000.1		CP012143.1	SN296	NGWE00000000.1	S1	JACDQB000000000.1
SN59	AZOP00000000.1		CP012137.1	SN297	NGWC00000000.1	S2	JACDQA000000000.1
SN60	AZOQ00000000.1	SN208	CP012138.1	SN298	NGWD00000000.1	S3	JAAGAS000000000.1
SN61	AZOR00000000.1		CP012139.1	SN299	NGWF00000000.1	S4	JAAGAR000000000.1
SN62	AZOS00000000.1		CP020336.1	SN300	NGVY00000000.1	S5	JACDPZ000000000.1
SN63	AZOT00000000.1	SN209	CP020337.1	SN301	NMXW00000000.1	S6	JAAGAQ000000000.1
SN64	AZOU00000000.1		CP020338.1	SN302	NMXX00000000.1	S7	JAAGAP000000000.1
SN65	AZOV00000000.1		CP020339.1	SN303	NMXZ00000000.1	S8	JAAGAO000000000.1
SN66	AZOW00000000.1	SN210	CP020340.1	SN304	NMXQ00000000.1	S9	JACDPY000000000.1
SN67	AZOX00000000.1		CP020341.1	SN305	NMXV00000000.1	S10	JACDPX000000000.1
SN68	AZOY00000000.1		CP020342.1	SN306	NMXP00000000.1	S11	JAAGAN000000000.1
SN69	AZOZ00000000.1	SN211	CP020343.1	SN307	NMXO00000000.1	S12	JAAGAM000000000.1
SN70	AZPA00000000.1		CP020344.1	SN308	PDXV00000000.1	S13	JACDPW000000000.1
SN71	AZPB00000000.1		CP020086.1	SN309	PDXU00000000.1	S14	JACDPV000000000.1
SN72	AZPC00000000.1	SN212	CP020087.1	SN310	PDXT00000000.1	S15	JAAGAL000000000.1
SN73	AZPD00000000.1		CP020088.1	SN311	PDXS00000000.1	S16	JACDPU000000000.1
SN74	AZPE00000000.1	SN213	CP020753.1	SN312	NXMF00000000.1	S17	JACDPT000000000.1
SN75	AZPF00000000.1		CP034060.1	SN313	NXME00000000.1	S18	JACDPS000000000.1
SN76	AZPH00000000.1		CP034059.1	SN314	PUHJ00000000.1	S19	JAAGAK000000000.1
SN77	AZPK00000000.1	SN225	CP034061.1	SN315	PUHK00000000.1	S20	JAAGAJ000000000.1
SN78	AZPM00000000.1		CP034062.1	SN317	UGYQ00000000.1	S21	JACDPR000000000.1
SN79	AZPN00000000.1		CP034063.1	SN319	UGYR00000000.1	S22	JAAGAI000000000.1

SN80	AZPP00000000.1		CP034064.1	SN322	UDYE00000000.1	S23	JAAGAH000000000.1
SN81	AZPQ00000000.1		CP034065.1	SN323	AZPG00000000.1	S24	JAAGAG000000000.1
SN82	AZPT00000000.1		CP030915.1	SN324	AZPI00000000.1	S25	JAAGAF000000000.1
SN83	AZPX00000000.1	SN229	CP030916.1	SN325	AZPJ00000000.1	S26	JAAGAE000000000.1
SN84	AZPZ00000000.1		CP030917.1	SN326	AZPL00000000.1	S27	JAAGAD000000000.1
SN85	AZQB00000000.1	SN230	AGNM00000000.1	SN327	AZPO00000000.1	S28	JACDQC000000000.1
SN86	AZQE00000000.1	SN232	ADUV00000000.1	SN328	AZPR00000000.1	S29	JAAGAC000000000.1

Table S3. General genomic characteristics of the 26 *stx* positive and three *stx* negative *S. flexneri* strains from Alberta.

**Strain name**	**S1**	**S2**	**S3**	**S4**	**S5**	**S6**	**S7**	**S8**	**S9**	**S10**
Genome size (Mb)	4.6	4.6	4.6	4.6	4.6	4.6	4.6	4.6	4.6	4.6
GC (%)	50.39	50.38	50.38	50.38	50.39	50.42	50.39	50.39	50.4	50.4
Average coverage	159.2	77.3	140.3	113	242.3	107.1	141	88.5	107.9	170.3
Genes (coding)	4615	4682	4653	4617	4681	4627	4683	4687	4656	4674
tRNA number	94	87	88	88	96	78	90	88	92	91
Strain name	S11	S12	S13	S14	S15	S16	S17	S18	S19	S20
Genome size (Mb)	4.6	4.6	4.6	4.6	4.6	4.6	4.6	4.6	4.6	4.6
GC (%)	50.39	50.39	50.39	50.38	50.44	50.41	50.41	50.41	50.42	50.41
Average coverage	82.4	113.5	127.6	97.1	87.6	120.1	98.9	138.9	49.3	163.9
Genes (coding)	4694	4679	4684	4695	4678	4580	4573	4603	4708	4669
tRNA number	90	89	88	87	89	89	91	88	89	91
Strain name	S21	S22	S23	S24	S25	S26	S27	S28	S29	
Genome size (Mb)	4.6	4.6	4.6	4.6	4.6	4.6	4.5	4.5	4.6	
GC (%)	50.4	50.39	50.38	50.39	50.4	50.4	50.74	50.75	50.6	
Average coverage	170.6	147.8	139.3	126.9	102.7	101.4	71.7	72.9	184.2	
Genes (coding)	4599	4643	4738	4704	4732	4681	4393	4483	4685	
tRNA number	91	90	95	80	92	90	79	79	85	

Table S4. SNP differences based on the core genome of the 26 *stx* positive *S. flexneri* strains from Alberta.

Strain	S13	S11	S14	S10	S15	S2	S23	S16	S17	S3	S22	S21	S19	S20	S18	S1	S6	S5	S8	S9	S4	S24	S25	S26	S7
S11	91																								
S14	70	101																							
S10	82	61	112																						
S15	108	113	114	104																					
S2	86	75	92	82	124																				
S23	108	119	132	96	122	124																			
S16	123	118	117	127	53	107	149																		
S17	105	102	133	115	59	117	141	76																	
S3	102	91	90	82	122	64	106	109	127																
S22	104	85	102	90	106	98	96	119	123	76															
S21	113	124	139	113	73	91	161	62	58	113	147														
S19	101	120	133	93	67	93	131	72	66	105	117	66													
S20	120	131	144	118	56	138	116	69	71	130	120	77	71												
S18	124	105	112	112	42	112	130	51	63	114	98	69	59	58											
S1	49	78	73	85	107	75	115	116	106	101	89	114	94	115	87										
S6	99	126	137	85	89	121	127	110	98	121	131	100	80	93	109	112									
S5	70	83	64	84	104	98	94	121	119	74	80	137	111	106	100	59	109								
S8	94	57	82	66	104	78	114	111	117	78	82	129	115	132	98	85	125	84							
S9	48	83	74	86	104	90	122	123	111	102	82	131	103	140	100	45	125	58	92						
S4	78	31	94	60	108	48	108	117	95	84	78	113	95	116	90	53	115	78	52	72					
S24	101	102	123	93	135	97	137	154	130	113	105	142	136	145	147	110	124	121	115	123	93				
S25	105	88	127	105	127	101	129	140	134	113	87	146	134	145	123	90	136	101	103	97	81	46			
S26	108	125	138	106	144	88	156	145	141	112	120	117	135	170	152	115	133	142	108	132	110	51	61		
S7	78	53	84	60	92	82	106	117	105	80	92	119	123	108	102	81	113	88	38	102	48	85	101	112	
S12	74	53	100	58	112	64	120	109	125	88	92	109	101	134	110	71	111	98	52	82	44	109	93	104	62

Table S5. Pairwise genome similarity 26 *stx*1 positive and 3 *stx*1 negative *S. flexneri* strains.

**Strain Name**	**Similarity**#	**Strain Name**	**Similarity**#
S2	99.5%	S16	99.3%
S3	99.7%	S17	99.3%
S4	99.7%	S18	99.3%
S5	99.7%	S19	99.3%
S6	99.3%	S20	99.3%
S7	99.6%	S21	99.4%
S8	99.7%	S22	99.7%
S9	99.5%	S23	99.5%
S10	99.7%	S24	99.6%
S11	99.7%	S25	99.6%
S12	99.6%	S26	99.6%
S13	99.4%	S27*	82.3%
S14	99.5%	S28*	82.5%
S15	99.3%	S29*	93.4%

# - The pairwise similarity is relative to *stx*1 positive *S. flexneri* strain S1

* - *stx*1 negative *S. flexneri* strains

Table S6. SNP differences based on the core genome of 55 *stx* positive *S. flexneri* strains.

Strain names	SN259	S13	S11	SN20	SN349	SN22	S15	S17	S3	S22	S19	S20	SN4	S1	SN8	SN6	S5	SN267	S9	SN269	SN262	S24	SN315	SN351	S26	SN260	S7	S12	SN21	SN23	S14	S10	SN352	SN19	S2	S23	S16	S21	SN264	SN5	SN341	SN339	S18	S6	S8	SN343	SN314	SN263	SN268	S4	SN7	SN261	SN18	S25
S13	39																																																				
S11	43	38																																																			
SN20	38	55	59																																																		
SN349	60	55	53	76																																																	
SN22	38	56	60	51	75																																																
S15	27	44	48	39	65	18																																															
S17	31	48	52	43	69	22	10																																														
S3	32	27	25	48	42	49	37	41																																													
S22	41	36	34	57	33	58	46	50	23																																												
S19	27	44	44	39	65	18	4	10	37	46																																											
S20	36	53	55	48	74	27	15	11	46	55	13																																										
SN4	29	46	50	23	67	42	30	34	39	48	30	39																																									
S1	36	5	35	52	52	53	41	45	24	33	41	50	43																																								
SN8	47	42	40	63	57	64	52	56	29	38	52	61	54	39																																							
SN6	10	49	53	48	70	48	37	41	42	51	37	46	39	46	57																																						
S5	40	9	37	56	56	57	45	49	28	37	43	52	47	6	43	50																																					
SN267	61	56	54	77	53	78	66	70	43	34	66	75	68	53	58	71	57																																				
S9	42	5	41	58	58	59	47	51	30	37	47	56	49	8	45	52	10	59																																			
SN269	67	62	60	83	27	84	72	76	49	40	72	81	74	59	64	77	63	60	65																																		
SN262	79	92	96	95	113	96	84	88	85	94	84	93	86	89	100	89	93	114	95	120																																	
S24	61	56	54	77	21	78	66	70	43	32	66	75	68	53	58	71	57	54	57	28	114																																
SN315	60	55	53	76	52	77	65	69	42	33	65	74	67	52	57	70	56	13	58	59	113	53																															
SN351	59	54	52	75	31	76	64	68	41	32	64	73	66	51	56	69	55	52	57	38	112	32	51																														
S26	60	55	53	76	20	77	65	69	42	33	65	74	67	52	57	70	56	53	58	27	113	1	52	31																													
SN260	32	49	53	44	70	41	29	33	42	51	29	38	35	46	57	42	50	71	52	77	89	71	70	69	70																												
S7	36	31	9	52	46	53	41	45	18	27	41	50	43	28	33	46	32	47	34	53	89	47	46	45	46	46																											
S12	40	35	13	56	50	57	45	49	22	31	45	54	47	32	37	50	36	51	38	57	93	51	50	49	50	50	6																										
SN21	95	90	88	111	67	112	100	104	77	68	100	109	102	87	92	105	91	88	93	74	148	68	87	58	67	105	81	85																									
SN23	67	79	84	83	101	84	72	76	73	82	72	81	74	76	88	77	80	102	82	108	120	102	101	100	101	77	77	81	136																								
S14	41	2	40	57	57	58	46	50	29	38	46	55	48	7	44	51	11	58	7	64	94	58	57	56	57	51	33	37	92	81																							
S10	39	34	6	55	49	56	44	48	21	30	44	53	46	31	36	49	35	50	37	56	92	50	49	48	49	49	5	9	84	80	36																						
SN352	46	41	45	62	62	63	51	55	34	43	51	60	53	38	49	56	42	63	44	69	99	63	62	61	62	56	38	42	97	87	43	41																					
SN19	59	54	52	75	19	76	64	68	41	32	64	73	66	51	56	69	55	52	57	26	112	20	51	30	19	69	45	49	66	100	56	48	61																				
S2	34	29	27	50	44	51	39	43	16	25	39	48	41	26	31	44	30	45	32	51	87	45	44	43	44	44	20	24	79	75	31	23	36	43																			
S23	57	52	46	73	67	74	62	66	39	48	58	69	64	49	54	67	51	68	55	74	110	68	67	66	67	67	43	47	102	98	54	46	59	66	41																		
S16	29	46	50	41	67	20	8	4	39	48	8	9	32	43	54	39	47	68	49	74	86	68	67	66	67	31	43	47	102	74	48	46	53	66	41	64																	
S21	32	49	53	44	70	23	11	7	42	51	11	10	35	46	57	42	50	71	52	77	89	71	70	69	70	34	46	50	105	77	51	49	56	69	44	67	5																
SN264	57	52	50	73	17	74	62	66	39	30	62	71	64	49	54	67	53	50	55	24	110	18	49	28	17	67	43	47	64	98	54	46	59	16	41	64	64	67															
SN5	32	49	53	44	70	23	9	15	42	51	9	20	35	46	57	42	50	71	52	77	89	71	70	69	70	34	46	50	105	77	51	49	56	69	44	67	13	16	67														
SN341	63	58	56	79	11	78	68	72	45	36	68	77	70	55	60	73	59	56	61	30	114	24	55	34	23	73	49	53	70	104	60	52	65	22	47	70	70	73	20	73													
SN339	116	133	137	102	154	129	117	121	126	135	117	126	101	130	141	126	134	155	136	161	173	155	154	153	154	122	130	134	189	161	135	133	138	153	128	151	119	122	151	120	157												
S18	26	43	47	38	64	17	5	9	36	45	5	14	29	40	51	36	44	65	46	71	83	65	64	63	64	28	40	44	99	71	45	43	50	63	38	61	7	10	61	10	67	116											
S6	33	50	52	45	71	46	34	38	43	52	32	41	36	47	58	43	49	72	53	78	90	72	71	70	71	39	47	51	106	78	52	50	57	70	45	66	36	39	68	39	74	123	33										
S8	39	34	12	55	49	56	44	48	21	30	44	53	46	31	36	49	35	50	37	56	92	50	49	48	49	49	3	9	84	80	36	8	41	48	23	46	46	49	46	49	52	133	43	50									
SN343	61	56	54	77	33	78	66	70	43	34	66	75	68	53	58	71	57	54	59	40	114	34	53	24	33	71	47	51	60	102	58	50	57	32	45	68	68	71	30	71	36	153	65	72	50								
SN314	71	88	92	83	109	78	66	70	81	90	66	75	74	85	96	81	89	110	91	116	128	110	109	108	109	73	85	89	144	116	90	88	95	108	83	106	68	71	106	71	112	161	65	78	88	110							
SN263	44	39	17	60	54	61	49	53	26	35	49	58	51	36	41	54	40	55	42	61	97	55	54	53	54	54	10	14	89	85	41	13	46	53	28	51	51	54	51	54	57	138	48	55	13	55	93						
SN268	52	47	45	68	12	69	57	61	34	25	57	66	59	44	49	62	48	45	50	19	105	13	44	23	12	62	38	42	59	93	49	41	54	11	36	59	59	62	7	62	15	146	56	63	41	25	101	46					
S4	33	28	10	49	43	50	38	42	15	24	38	47	40	25	30	43	29	44	31	50	86	44	43	42	43	43	3	7	78	74	30	6	35	42	17	40	40	43	40	43	46	127	37	44	6	44	82	11	35				
SN7	45	58	62	61	79	62	50	54	51	60	50	59	52	55	66	55	59	80	61	86	98	80	79	78	79	55	55	59	114	86	60	58	65	78	53	76	52	55	76	55	82	139	49	56	58	80	94	63	71	52			
SN261	44	39	37	60	36	61	49	53	26	17	49	58	51	36	41	54	40	37	42	43	97	37	36	35	36	54	30	34	71	85	41	33	46	35	28	51	51	54	33	54	39	138	48	55	33	37	93	38	28	27	63		
SN18	53	50	54	69	71	70	58	62	43	52	58	67	60	47	58	63	51	72	53	78	106	72	71	70	71	63	47	51	106	94	52	50	57	70	45	68	60	63	68	63	74	147	57	64	50	72	102	55	63	44	72	55	
S25	60	55	53	76	20	77	65	69	42	33	65	74	67	52	57	70	56	53	58	27	113	1	52	31	0	70	46	50	67	101	57	49	62	19	44	67	67	70	17	70	23	154	64	71	49	33	109	54	12	43	79	36	71	

SN9	28	45	49	40	66	19	7	3	38	47	7	8	31	42	53	38	46	67	48	73	85	67	66	65	66	30	42	46	101	73	47	45	52	65	40	63	1	4	63	12	69	118	6	35	45	67	67	50	58	39	51	50	59	66

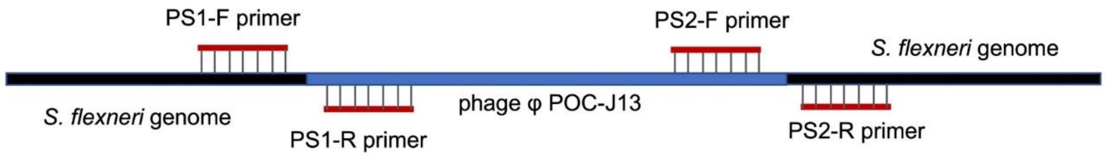

Figure S1. Location of primers for confirming the specific insertion of phage φPOC-J13 in the chromosome of *S. flexneri*.

Reference

1. Perelle, S.; Dilasser, F.; Grout, J.; Fach, P. Detection by 5'-nuclease PCR of Shiga-toxin producing *Escherichia coli* O26, O55, O91, O103, O111, O113, O145 and O157:H7, associated with the world's most frequent clinical cases. *Mol Cell Probes* **2004**, *18*, 185-192, doi:10.1016/j.mcp.2003.12.004.
2. Scheutz, F.; Teel, L.D.; Beutin, L.; Pierard, D.; Buvens, G.; Karch, H.; Mellmann, A.; Caprioli, A.; Tozzoli, R.; Morabito, S.; et al. Multicenter evaluation of a sequence-based protocol for subtyping Shiga toxins and standardizing Stx nomenclature. *J Clin Microbiol* **2012**, *50*, 2951-2963, doi:10.1128/JCM.00860-12.
